# Anti-oxidant and anti-apoptotic effects of gamma-oryzanol on male reproductive function in chronic restraint stress in rats

**DOI:** 10.22038/AJP.2024.24819

**Published:** 2025

**Authors:** Mahsan Alizadeh, Sina Moshtagh, Shahabaddin Abdolalizadeh Amir, Mostafa Jeddi, Sepehr Tahmasebzadeh, Ghazal Radman, Amin Bagheri, Yasin Bagheri, Nazanin Shahabinejad

**Affiliations:** 1 *Department of Clinical Sciences, Faculty of Veterinary Medicine, Tabriz Medical Sciences, Islamic Azad University, Tabriz, Iran*; 2 *Department of Clinical Science, Theriogenology Section, School of Veterinary Medicine, Razi University, Kermanshah, Iran*; 3 *Molecular Medicine Research Center, Hormozgan Health Institute, Hormozgan University of Medical Sciences, Bandar Abbas, Iran*; 4 *Department of Urology, Sina Hospital, Tabriz University of Medical Sciences,* *Tabriz, Iran*; 5 *Kidney Research Centre, Tabriz University of Medical Sciences,* *Tabriz, Iran*; 6 *Student Research Committee, Kerman University of Medical Sciences, Kerman, Iran *

**Keywords:** Gamma-oryzanol, Chronic restraint stress, Sperm parameters, Apoptosis, Oxidative stress

## Abstract

**Objective:**

This study investigates the effects of GO on depressive behaviors and reproductive parameters in rats exposed to CRS.

**Materials and Methods:**

Rats were divided into three groups: sham, CRS-control, and CRS-GO. Behavioral assessments using the SPT and measurements of body and testes weights were conducted. Reproductive potential was evaluated by ELISA for testosterone, LH, and FSH levels, as well as sperm characteristics. Oxidative stress levels were assessed through MDA measurements and antioxidant enzyme activities. Additionally, apoptotic and signaling pathway proteins were analyzed by measuring cleaved caspase-3 and Nrf-2 levels in the testes using western blot analysis.

**Results:**

GO mitigated depressive behaviors and reduced serum corticosterone levels compared to the CRS-control group. GO increased testosterone, LH, and FSH levels and improved sperm parameters. GO supplementation reduced MDA levels and enhanced antioxidant enzyme activities compared to the CRS-control group. The analysis showed that GO down-regulated cleaved caspase-3 levels and up-regulated Nrf-2 protein levels in the testes of CRS rats compared to the CRS-control group.

**Conclusion:**

The administration of GO treatment can contribute to the recovery from male reproductive complications by regulating oxidative stress and apoptotic pathways.

## Introduction

Stress is acknowledged as a critical factor with the potential to adversely impact various facets of human life. It is defined as any uncomfortable emotional experience accompanied by predictable physiological, biochemical, and behavioral alterations (Baum, 1999). A direct relationship has been observed between stress and exposure to elevated levels of stress hormones, involving various organs and contributing to the development of various diseases (Vazquez-Palacios Gand and Velazquez-Moctezuma, 2000). Furthermore, exposure to unpredictable environmental stress is considered a primary indicator of risk and severity in neuropsychiatric diseases such as depression, anxiety, and post-traumatic stress disorder (PTSD) (Anderson et al., 2019). The adverse effects of stress can disrupt the maintenance of homeostasis (Cavigelli et al., 2018). 

Stressors can be categorized based on factors such as duration and intensity, resulting in acute (single and short-duration stress with high intensity) and chronic (long-term and repeated stress with low or moderate intensity) stress (Weissman et al., 2007; Pacak, 2000). Animal models have been developed to understand the pathophysiology of stress and its consequences better since human samples are limited and present a variety of unpredictable circumstances (Wang et al., 2017; Golbidi et al., 2018). Restraint immobilization (IMO) is a common procedure employed to induce a combination of physical and psychological stressors by limiting free movement through placing the rat in a restricted space and isolating it from its group (Maghsoudi et al., 2014). Long-term exposure to physical or mental stress induces structural and neurochemical alterations in brain regions regulating mood and cognition, such as the hippocampus and prefrontal cortex (James et al., 2023). Additionally, the hypothalamic-pituitary-adrenal (HPA) axis (involved in chronic stress) and the sympathetic nervous system (involved in acute stress) are two neuroendocrine systems contributing to responses in coping with stress-related conditions (Charmandari et al., 2005; Moberg, 1987). These responses affect the secretion of hormones such as adrenocorticotropic hormone (ACTH), corticotropin-releasing factor, luteinizing hormone (LH), and adrenal corticosteroids (Plant, 2015). HPA axis dysfunction, resulting in damage to brain tissue and synaptic neuron plasticity, may lead to the development and progression of depressive disorders (Schumacher et al., 2019). Some studies suggest a correlation between high levels of reactive oxygen species (ROS), lipid peroxidation, and protein oxidation with depressive symptoms (Black, 2015). Additionally, mitochondria, the main energy source of cells, regulate ROS and apoptosis. A direct relationship has been reported between mitochondrial disease and depression (Lopresti, 2019). Consequently, neurobehavioral disorders triggered by chronic stress are associated with mitochondrial dysfunction and oxidative stress (Lucca et al., 2009). 

Moreover, reproduction plays a crucial role in population perpetuation, controlled by the hypothalamic-pituitary-gonadal (HPG) axis, making male reproductive health a significant aspect of public health (Iranloye et al., 2013). Chronic stress negatively affects sperm concentration, morphology, motility, and production, testosterone hormone levels, and testicular weights (Clarke et al., 1999; Demirci and Sahin, 2019; Hari Priya and Reddy, 2021). Restraint stress can result in reproductive dysfunction, including low testosterone secretion, decreased sexual motivation and spermatogenesis, delayed testicular maturation in male rats, prolonged intromission, less ejaculation, ejaculation latency, adverse effects on semen quality and sperm morphology and concentration, decreased testes weight, and germ cell degeneration (Almeida et al., 2000; Grønli et al., 2005; Bonde et al., 1998; Kholkute and Udupa, 1979). 

In light of the available evidence, male infertility is notably impacted by oxidative stress stemming from both physical and psychosocial stressors. Fundamental mechanisms include the recruitment of leukocytes triggered by pro-inflammatory signals, the generation of ROS from sperm mitochondria, and the dysregulation of pivotal enzymes such as NADPH and amino acid oxidases, are crucial for sperm capacitation (Aitken et al., 2022). The influence of excessive ROS production on oxidative stress during germ cell development hinges on maintaining equilibrium between pro- and antioxidant forces (Aitken et al., 2022). 

Consequently, it is recommended to employ natural antioxidants to counteract the detrimental effects of stress (Adewoyin et al., 2017; Cilio et al., 2022). For instance, dietary antioxidant supplementation is suggested to yield potential enhancements in sperm quality, encompassing the mitigation of oxidative stress-induced damage and improvements in hormone synthesis, sperm concentration, motility, and morphology (Cilio et al., 2022). 

Gamma-oryzanol (GO) has garnered attention due to its various health-promoting functions, with rice bran being a primary source (Marić et al., 1996). Other sources of GO include wheat, maize, oats, tomatoes, asparagus, berries, peas, olives, fruits, vegetables, and several other foods (Akter et al., 2020). Reports indicate that GO has no major side effects (Moon et al., 2017). Numerous studies have elucidated the health-beneficial properties of GO, including improving plasma lipid patterns, inhibiting platelet aggregation, antioxidant functions, antidiabetic and allergic reactions, deterring tumor promotion, hyperlipidemia, fatty liver, hypercalciuria, renal damage, heart disease, treating inflammation, nervousness, and menopause disorders, and increasing muscle mass (Islam et al., 2011; Araujo et al., 2015; Chotimarkorn and Ushio, 2008; Murase and Iishima, 1963; Bonner et al., 1990). Additionally, some evidence emphasizes the effects of GO on the brain and its role in modulating the function of the hypothalamus and anterior pituitary gland (Xu and Godber, 1999). GO contains trans-ferulic acid esters and phytosterols (Escobar, 2019). Phytosterols present in GO play a critical role in the synthesis of essential biomolecules such as sex hormones (Xu and Godber, 1999), resulting in the production of steroidal hormones (Deuster et al., 2004). However, some studies have defined GO as a parameter to increase the levels of testosterone, growth hormone, and other muscle-building or anabolic hormones, as well as treating the symptoms related to menopause (Murase and Iishima, 1963). 

Furthermore, recent scholarly investigations have directed attention towards elucidating the antioxidant properties of GO (Minatel et al., 2016). Bumrungpert et al. posit that rice bran oil containing gamma-oryzanol could potentially assume a pivotal role in diminishing cardiovascular disease risk factors. This is postulated to occur through the reduction of low-density lipoprotein levels and the augmentation of antioxidant capacity, particularly in hyperlipidemic subjects (Bumrungpert et al., 2019). Expanding on the multifaceted roles of GO, Rungratanawanich et al. extensively expound on its contributions to improving brain function. These contributions encompass the modulation of synaptic plasticity and neuronal trafficking, influence on mitochondria and energy metabolism, and provision of neuroprotection coupled with antioxidant activity (Rungratanawanich et al., 2019). Moreover, Klongpityapong et al. shed light on the impact of GO on human prostate cancer cells, elucidating its influence on the down-regulation of specific antioxidant genes, including catalase (CAT) and glutathione peroxidase (GPX) (Klongpityapong et al., 2013). 

Following our investigations, the protective effects of GO on male reproductive function in rats undergoing chronic restraint stress (CRS) have not been studied thoroughly yet. Therefore, the purpose of the present study was to determine the protective effects of GO on male reproductive parameters and depressive-like behaviors in restraint-stressed rats.

## Materials and Methods

### Characteristics and materials procurement

GO, a crystalline and odorless oil-soluble powder, exhibits a white or yellowish-white color with a purity of 99%. The GO utilized in this study was sourced from Syna Vison Co. (Iran). Essential antibodies for western blotting were procured from Santa Cruz Biotechnology, Inc. (Texas, USA). For oxidative stress factors and hormonal assessment, commercial test kits from BiocoreZellbio (Germany) were employed. Rats for the study were supplied by the Pasteur Institute (Tehran, Iran).

### Animal model and study design

All procedures adhered to the ARRIVE guidelines and criteria (Percie du Sert et al., 2020). Male adult Wistar rats (230±20 g), totaling 24, were acquired from the Pasteur Institute (Tehran, Iran). The sample size, following a previous model (Nouri et al., 2019), was determined for the experiment. Initially housed in a controlled environment, the rats experienced a 10-day adaptation period with a temperature of 22±2°C, humidity of 50±5%, and a 12/12 hr light-dark cycle. Water and food were provided *ad libitum* during this period. A total of 24 rats were randomly allocated into three experimental groups. The first group served as the control (sham) group (N=8), receiving normal saline (NS) orally (2 ml/kg) without exposure to stress. The second group was the chronic restraint stress (CRS) control group (N=8), receiving NS orally (2 ml/kg) alongside exposure to CRS. The third group, also exposed to CRS (N = 8), received GO treatment (100 mg/kg body weight) orally via gavage (Phannasorn et al., 2021). The GO treatment was administered as a single dose prior to the restraint stress. Qualitative and quantitative assessments were performed upon completion of the stress exposure period.

### Ethical statement

All rats were treated according to high ethical and scientific standards, following the Guideline for the Care and Use of Laboratory Animals in Iran (Ahmadi-Noorbakhsh et al., 2021). Protocols were implemented in compliance with relevant guidelines and regulations (IR.TBZMED.AEC.1403.005). 

### Inducing CRS rats

Rats in both the CRS-Control and CRS-GO groups were subjected to restraint stress, consistent with prior studies (Bagheri et al., 2021a; Bagheri et al., 2020). Rats were confined in a ventilated container for two hours daily, without access to water or food, for 21 consecutive days, from 10:00-12:00 a.m. Control rats were kept in their cages without access to water or food during the restraint stress protocol. 

### Sucrose preferences test (SPT)

Behavioral assessments were conducted at the end of 21 days of stress exposure using the Sucrose Preferences Test (SPT). Twenty-four hours before behavioral assessment, two bottles of 1% sucrose solution were placed in the animals' cages. The next day, one of them was replaced with a bottle of tap water. Also, the quantity of sucrose solution and water intake was recorded, and the preference was measured based on this formula: (the weight of sucrose solution intake/the weight of total solution intake) × 100%.

### Sample collection

The animals underwent anesthesia using intraperitoneal injection of ketamine and xylazine (90 and 10 mg/kg, respectively) 24 hr post-behavioral testing (Salehpour et al., 2019). Subsequently, blood samples were obtained through cardiac puncture, and collected in sampling vials for hormone and parameter assessment. After centrifugation at 3000 rpm for 10 min at 4°C, serum was extracted and stored at -20°C or lower. 

Testes were meticulously removed, rinsed in 0.9% normal saline, weighed, and homogenized in ice-cold phosphate buffer (pH 7.4). Finally, the cauda epididymis (approximately 1 cm of the epididymal tail) was excised for sperm storage evaluation.

### Hormonal profile

Serum levels of corticosterone (CORT), testosterone, luteinizing hormone (LH), and follicle-stimulating hormone (FSH) were quantified using enzyme-linked immunosorbent assay (ELISA) kits, following the manufacturer's guidelines. The DBC ELISA kit, with specific catalog numbers and sensitivities (CORT: ab108821, 0.3 ng/ml; testosterone: CAN-TE-250, 0.022 ng/ml; LH: CAN-LH-4040, 0.2 IU/L; FSH: CAN-FSH-4060, 1 IU/L), was employed for hormone measurement. 

### Sperm parameters

 The weight of the right and left testes in each experimental rat was recorded. Subsequently, the cauda epididymis was dissected to examine sperm storage. The epididymal contents were drained, weighed, and then incubated in a 2 ml culture medium (Ham's F10) containing 0.5% serum albumin at 37°C. The solution was periodically shaken after the addition of a balanced buffer (Khaksar et al., 2017). 

### Sperm motility

Sperm motility was subjectively assessed by visual estimation, performed consistently by the same observer throughout the study. Progressive motility was quantified as the percentage of sperm cells exhibiting forward movement. To evaluate motile sperm, a drop of sperm suspension was placed on a glass slide, covered with a coverslip, and examined under a light microscope (Olympus Life Sciences, Japan) at 400x magnification (Chanapiwat P and Kaeoket K, 2015). 

### Sperm count

 A 10% formalin in phosphate buffered saline solution was used to dilute the sperm suspension. Subsequently, 10 μl of the diluted solution was transferred to the Neubauer hemocytometer, and the total sperm count within the small square was calculated (Khaksar et al., 2017). 

### Sperm viability

Sperm viability was determined through Eosin Y staining (5% in saline). A mixture of 40 µl of sperm suspension with 10 ml of Eosin was placed on a glass slide and observed under a light microscope (X400). Live sperm remained unstained, while dead sperm exhibited red or pink coloration. Viability is expressed as a percentage, with 200 sperm counted from each sample in ten randomly selected fields of vision (Khaksar et al., 2017).

### Testicular tissue non-enzymatic and enzymatic antioxidant assays

Malondialdehyde (MDA) levels were quantified through the thiobarbituric acid reactive substances (TBARS) method and the Uchiyama technique, with absorbance readings at 532 nm (Uchiyama and Mihara, 1978). Concurrently, superoxide dismutase (SOD) activity in testis tissue was assessed via the *Marklund* and *Marklund* method, relying on the prevention of pyrogallol autoxidation at pH 8 (Marklund and Marklund, 1974). Catalase (CAT) activity was determined using the Claiborne method, involving H2O2, and measured at 240 nm (Claiborne, 1985). Additionally, glutathione peroxidase (GPx) activity was evaluated based on the Paglia and Valentine method (Paglia and Valentine, 1967). To ensure accuracy, the levels of all these oxidative factors were determined using a commercial test kit (Germany: ZellBio GmbH).

### Western blot analysis

Western blotting was employed to assess cleaved-caspase3 and Nrf-2 levels. Testis tissues were dissolved in lysis buffer, and total protein was extracted, with supernatants collected for analysis. Total protein concentration was determined using the Bradford protein assay, and 12 mg/ml of each sample was loaded onto a 10% SDS-PAGE gel. Protein transfer to a polyvinylidene difluoride (PVDF) membrane was followed by blocking in 5% skim milk at room temperature. Subsequently, western blot analysis was conducted using primary antibodies (incubated overnight at 4°C) against cleaved-caspase (SC-56052; Santa Cruz, CA), caspase 3 (SC-7272, Santa Cruz Biotechnology, Santa Cruz, CA), Nrf2 ((A-10):sc365949, Santa Cruz Biotechnology, Santa Cruz, CA), and secondary antibodies (incubated for two hours at room temperature) conjugated with peroxidase anti-rabbit IgG-HRP (Cat. No. sc-2357) for enhanced chemiluminescence reaction. Protein band intensity was analyzed using Image J 1.6 software (Jafari et al., 2020). 

### Statistical analysis

The data are expressed as mean±standard error of the mean (SEM). Statistical analysis was conducted using IBM SPSS version 23, employing one-way ANOVA followed by Tukey's *post-hoc* multiple comparison tests to compare the experimental groups. P-value of less than 0.05 was considered statistically significant. 

## Results

### Improving the behavioral index analysis of immobilized rats by GO

**Figure 1:**
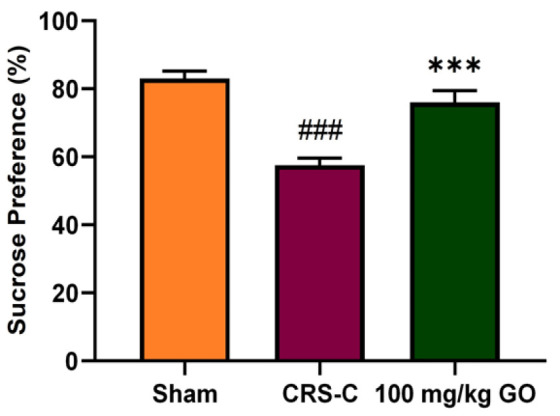
Effects of GO on the percentage of sucrose preference evaluated in the experimental groups after the restraint stress exposure. The data are presented as mean±SEM (N=8). ###p<0.001 vs. the sham group. ***p<0.001 vs. the CRS-C group. CRS-C: chronic restraint stress control; GO: gamma oryzanol.

As shown in Figure 1, the sucrose preference test was applied to assess anhedonia-like behavior, which is regarded as a classic component of depressive-like behavior in rats. The results represent that rats subjected to CRS consume significantly less sucrose compared with the sham group (p<0.001), indicating that 21-day CRS induces anhedonia in rats. But, GO prevented the development of anhedonia-like behavior in CRS-affected rats. CRS animals administered with 100 mg/kg GO showed a higher sucrose preference compared with the CRS-control rats (p<0.001), which is almost similar to the sham group.

### Effects of GO on the body and testis weights in immobilized rats

**Figure 2:**
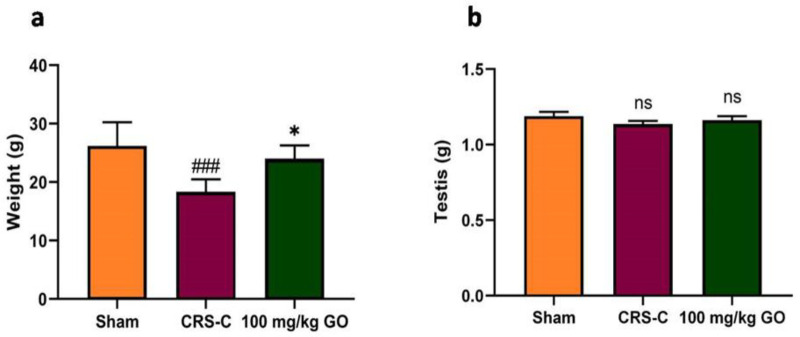
Effects of GO on (a) Body weight and (b) Testis weights in the experimental groups after restraint stress exposure. The data are shown as mean±SEM (N=8). ###p<0.001 vs. the sham group. *p<0.05 vs. the CRS-C group. CRS-C: chronic restraint stress control; GO: gamma oryzanol; ns: non-significant

Figure 2 illustrates the effects of GO on mean body weight and testicular weight. The one-way ANOVA analysis of the body weight of rats exposed to chronic immobilization stress exhibits significant body weight loss compared with the sham group (p<0.001). However, the body weight in rats increased significantly after GO administration (100 mg/kg) in CRS ones compared with CRS-control ones (p<0.05). Based on the analysis, no significant difference was observed in the mean testis weight among the study groups. Therefore, testicular tissue weight is not considered a reliable index to track the effect of immobilization stress in the rat model.

### Controlling serum corticosterone levels in immobilized rats by GO

**Figure 3:**
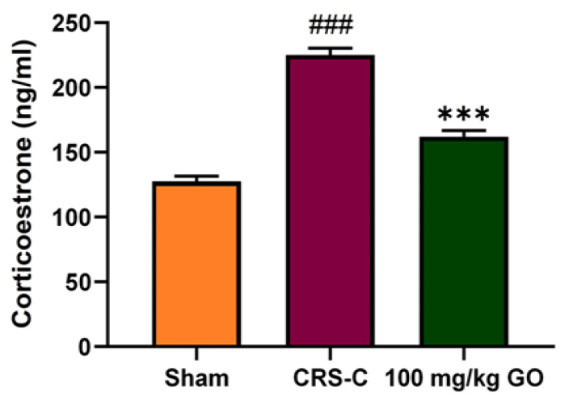
Effect of GO on serum corticosterone levels in the experimental groups after restraint stress exposure. The data are presented as mean±SEM (N=8). ###p<0.001 vs. the sham group. ***p<0.001 vs. the CRS-C group. CRS-C: chronic restraint stress control; GO: gamma oryzanol

Figure 3 demonstrates the effect of CRS induced by immobilization on plasma corticosterone levels. The results indicate a significant rise in plasma corticosterone levels in the CRS-control rats compared with the sham group (p<0.001), which decreased significantly following GO-treatment in doses of 100 mg/kg compared with the CRS-control group (p<0.001). 

### Improving serum levels of reproductive hormones in immobilized rats by GO

**Figure 4:**
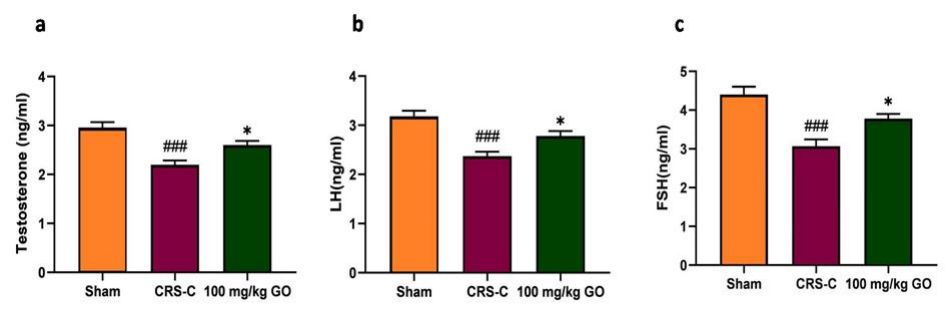
Effects of GO on the reproductive hormone levels in the experimental groups after restraint stress exposure. (a) Testosterone, (b) LH, and c. FSH. The data are shown as mean±SEM (N=8). ###p<0.001 vs. the sham group. *p<0.05 vs. the CRS-C group. CRS-C: chronic restraint stress control; GO: gamma oryzanol

Figure 4 illustrates the plasma concentrations of testosterone, LH, and FSH. A 21-day immobilization-induced stress exposure in rats resulted in a significant reduction in reproductive hormone levels compared with the sham rats (p<0.001 for testosterone, LH, and FSH). Notably, treatment with GO at 100 mg/kg demonstrated an elevation in serum reproductive hormone levels in rats subjected to CRS via immobilization when compared with the CRS-control group (p<0.05 for all three parameters). This effect suggests the potential of GO to mitigate the adverse impact of restraint stress on fertility hormones.

### Improving sperm parameters in immobilized rats by GO

**Figure 5:**
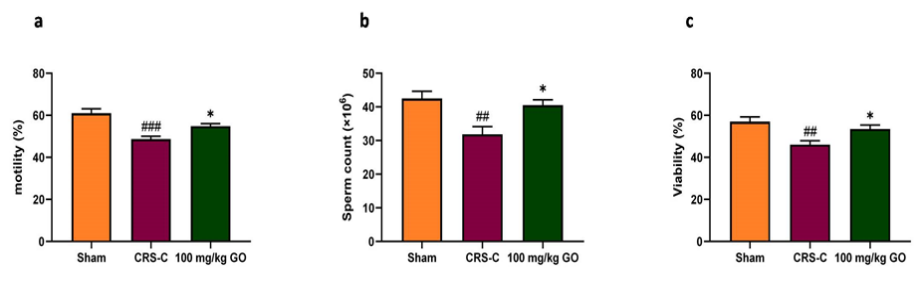
Effects of GO on sperm parameters in experimental groups after restraint stress exposure. (a) Percentage of sperm motility, (b) Sperm counts, and (c) Percentage of sperm viability. The data are presented as mean±SEM (N=8). ##p<0.01 and ###p<0.001 vs. the sham group. *p<0.05 vs. the CRS-C group. CRS-C: chronic restraint stress control; GO: gamma oryzanol

Figure 5 shows the percentage of sperm motility and viability, as well as mean sperm counts. The results were analyzed employing one-way ANOVA. Accordingly, sperm motility, sperm counts, and percentage of sperm viability reduced significantly after CRS compared with sham rats (p<0.001, p<0.01, and p<0.01, respectively). However, the oral administration of GO (100 mg/kg), along with CRS reverted the detrimental effects of immobilization stress on sperm parameters significantly and restored these parameters close to those of the sham rats (p<0.05 for all three parameters), resulting in minimizing the adverse influences of CRS on sperm quality.

### Modulating the levels of antioxidant and oxidative stress indicators in testis tissues of immobilized rats by GO

**Figure 6:**
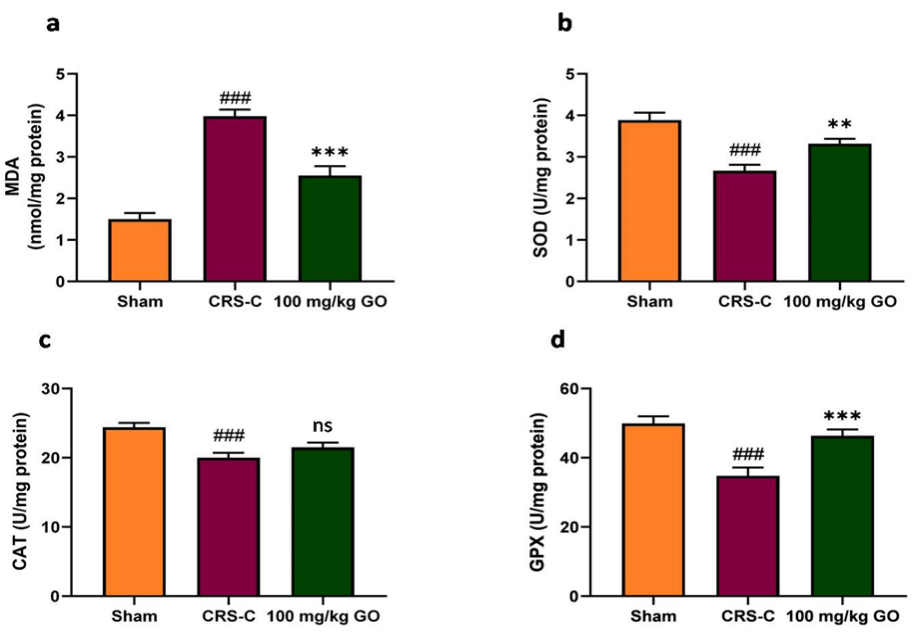
Effects of GO on the antioxidant levels of testicular tissue in the experimental groups after restraint stress exposure. (a) MDA, (b) SOD, (c) CAT, and (d) GPx. The data are shown as mean±SEM (N=8). ###p<0.001 vs. the sham group. **p<0.01 and ***p<0.001 vs. the CRS-C group. CRS-C: chronic restraint stress control; GO: gamma oryzanol; ns: non-significant

Figure 6 illustrates the variations in antioxidant levels and activity of testicular tissue. Rats subjected to immobilization-induced chronic stress showed a significant increase in MDA levels compared with the sham group (p<0.001). However, treating CRS rats with 100 mg/kg GO reduced MDA levels significantly compared with the CRS-control group (p<0.001). In addition, a significant decline was found in the levels of the enzymatic antioxidants SOD, CAT, and GPx in the CRS-control rats (p<0.001 for all of the three antioxidants) compared with CRS-control and sham ones, while treating with GO in the CRS rats increased the testicular SOD and GPx levels compared with the CRS-control groups (p<0.01 and p<0.001, respectively). Further, a slight yet non-significant rise was found in CAT levels in the CRS-treated group compared with the CRS-control one. Thus, administering GO could increase the level of enzymatic antioxidants compared with the CRS-control group. However, such values still lag below the sham levels, indicating that the oxidative function indicators in the testis tissues which mediate chronic stress immobilization are modified by the GO therapy. 

### Regulating testicular protein expression

**Figure 7:**
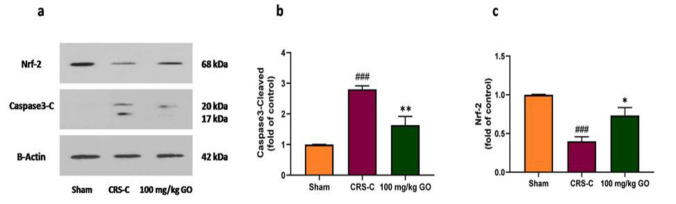
Effects of GO on protein expression in the testicular tissues of experimental groups after restraint stress exposure. (a) Representative images of protein expressions investigated by western blotting, (b) Caspase 3-cleaved, and c. Nrf-2. The data are shown as mean±SEM (N=3). ###p<0.001 vs. the sham group. *p<0.05 and **p<0.01 vs. the CRS-C group. CRS-C: chronic restraint stress control; GO: gamma oryzanol

Chronic RS influences the upregulation of cleaved caspase-3 and Nrf-2 protein levels in testicular tissues. Figure 7a demonstrates representative western blots. A significant increase is observed in protein expression of cleaved caspase-3, which is regarded as a key component of the mitochondrial-dependent apoptosis pathway in the CRS-control group compared with the sham one (p<0.001). However, daily GO treatments diminished the level of cleaved caspase-3 in the testis tissue at a dosage of 100 mg/kg (p<0.01) (Figure 7b).

To understand the link between the regulation of Nrf-2 protein expression and factors that control antioxidant protein expression, immobilized rats were given GO, and protein levels were examined. As Figure 7c displays, the expression of Nrf-2 was significantly lower in the testis tissue of the CRS-control group compared with the sham one (p<0.001). In addition, GO enhanced the level of Nrf-2 expression at 100 mg/kg in the testis tissue of the CRS rats compared with the CRS-control rats (p<0.05).

## Discussion

Overwhelming evidence supports the application of nutrition as a therapeutic strategy for many diseases or pathologic situations. The biologically active constituents of functional meals can be used to preserve body health through their antioxidative action (Lobo et al., 2010). Such bioactive components show a wide range of activities including anti-tumor (Raicht et al., 1980), anti-oxidant (Lin et al., 2020; Minatel et al., 2016; Wunjuntuk et al., 2016), anti-inflammatory (Lin et al., 2020), and anti-apoptotic (Bagheri et al., 2023; Minatel et al., 2016), as well as regulating the level of cholesterol (Berger et al., 2005), blood glucose (Kozuka et al., 2015), and body weight (Lin et al., 2020). Based on some studies, GO can play protective roles in various organs such as the kidneys, liver, heart, brain, and testis (Minatel et al., 2016; Bagheri et al., 2021b; Berger et al., 2005; Kozuka et al., 2015; Zaidi SKR and Banu; 2004). 

\

In this study, restraint stress was applied as the stressor to induce oxidative stress and apoptosis. The results indicated that exposure to restraint stress decreases body weight and levels of reproductive hormones significantly. In addition, a clear reduction was found in sperm motility, counts, and viability, as well as the percentages of sucrose preference. Furthermore, a decrease was observed in antioxidant enzyme activity including SOD, CAT, GPX, and Nrf-2, and an increase was found in cleaved caspase-3, level of MDA, and stress hormones. Previous studies in accordance with our results have reported higher levels of lipid peroxidation and lower antioxidant levels in testicular tissue due to restraint stress (Zainab et al., 2020; Karna et al., 2020). The results are consistent with those of previous studies, indicating that immobilization stress decreases testis weight, sperm count, and motility (Karna et al., 2020; Guo et al., 2017). In addition, alterations in sperm parameters and abnormal spermatogenesis may derive from a higher level of ROS formation, changes in the level of hormonal parameters, and atrophy of seminiferous tubules due to restraint stress (Karna et al., 2020; Arun. et al., 2016). Based on the results, exposure to restraint stress may lead to higher levels of oxidative stress and lower levels of antioxidant enzymes (Karna et al., 2020; Atif et al., 2008). Elevated levels of oxidative stress pose a direct risk to fertility by inducing oxidative stress, resulting in structural alterations at the cellular level. Additionally, these high ROS levels can exert an indirect impact by influencing the hypothalamic axes responsible for hormone release. Oxidative stress contributes to a reduction in male sex hormone levels, disrupting the delicate hormonal balance crucial for regulating male reproductive functions, ultimately leading to infertility (Darbandi et al., 2018). An elevation in caspase-3 as an apoptotic trigger and a reduction in Nrf-2 indicated an increase in testicular apoptosis in restraint-stressed rats, which is in line with the results of the previous studies (Karna et al., 2020; Arun. et al., 2016).

In view of the aforementioned, the present study aimed to delineate the protective effects of GO on male reproductive parameters in restraint-stressed rats. The results showed that GO promotes LH, FSH, and testosterone levels. Moreover, treatment with GO led to a significant enhancement in body weight, sperm numbers, sperm motility, sperm viability, and percentages of sucrose preference. Although improving the testis weight was not remarkable, our obtained results coincided with earlier reports that found GO to be an effective substance for male reproductive parameters recovery in rats under stress (Escobar et al., 2019). Improving sperm viability, sperm motility, and sperm concentration may be due to the antioxidant and antiapoptotic effects of the GO which were reported in this study and recent studies (Arun et al., 2016; Najafi et al., 2022).

Regulation of FSH, LH, and testosterone levels demonstrated that GO may affect gonadotropin-releasing hormone (GnRH) in the hypothalamus, subsequently influencing the function of the anterior pituitary gland to secrete LH and FSH. This ultimately leads to the recovery of testosterone production and spermatogenesis. In addition, the higher level of testosterone may be attributed to lower levels of glucocorticoids, leading to higher expression of testosterone hormone and enhanced LH-induced testicular secretion of testosterone (SAPOLSKY, 1985).

The SPT is considered a behavioral test based on rewards, which is applied as an anhedonia indicator (Kesyou et al., 2021). Based on the results, GO consumption increased sucrose preference, which can propose GO as an antidepressant. Such antidepressant features and increasing sucrose preferences following GO consumption were consistent with the results reported by other researchers (Arun et al., 2016; Akter et al., 2019). The anxiolytic effect may arise from the regulation of the corticosterone hormone and monoamine levels in the brain (Akter et al., 2019). 

GO treatment partially compensated for body weight loss, which is consistent with another piece of evidence (Akter et al., 2019). However, some other investigations reported that GO was not effective in body loss treatment (Araujo et al., 2021).

The results of this study, which are in line with those of the previous ones, represented that restraint stress can increase the oxidation capacity in different organs (Bandegi et al., 2014). An imbalance between the production of ROS and its removal by antioxidant mechanisms is among the characteristic features of oxidative stress (Das and Roychoudhury, 2014). The levels of MDA and activities of SOD, CAT, and GPx were assayed to study the protective effects of GO against oxidative damage induced by restraint stress. The results indicated that GO upregulated antioxidant enzyme activity and decreased MDA levels in the testis. Based on the results of some studies, ferulic acid and sterol moiety are responsible for the antioxidant properties of GO (Islam et al., 2011). Decreasing the level of corticosterone (Zafir and Banu, 2009), lowering the formation of ROS (WARD and TILL, 1990), and regulating the expression of Nrf-2 (Wang et al., 2020) may be related to an increase in the level of antioxidant enzymes. de Gomes et al. reviewed the protective effect of GO supplementation in acute liver failure due to acetaminophen usage and claimed that GO protected against the decrease in CAT, SOD, aminolevulinic acid dehydratase and GPx activities due to acetaminophen-induced acute liver failure (de Gomes et al., 2020). 

Corticosterone, the primary glucocorticoid in rodents, is influenced by stressful events such as restraint stress, leading to activation of the HPA axis. This activation involves an increase in CRH from the hypothalamus, triggering the secretion of ACTH from the anterior pituitary, ultimately resulting in the release of glucocorticoids from the adrenal cortex (Armario, 2006). Consistent with earlier research, our findings indicated that chronic restraint stress elevated corticosterone levels (Asada, 1999), attributed to stress-induced anxiety. The heightened corticosterone levels were linked to HPA stimulation (Moustafa, 2021). Interestingly, our results demonstrated that GO consumption decreased corticosterone levels by upregulating the central monoaminergic system in the amygdala, aligning with the anti-stress properties observed in previous studies (Asada, 1999). This suggests GO as a potential anti-stress agent, supporting existing literature.

Based on the analysis, GO downregulated the apoptosis-regulating genes of cleaved caspase-3 and up-regulated Nrf-2 in the restraint-stressed rats treated with GO significantly. Some other studies confirmed that GO downregulated pro-apoptotic proteins including caspase-3, caspase-9, and Bax (Meng et al., 2022; Najafi et al., 2022; de Gomes et al., 2020). Such regulation may derive from the reduction of cell apoptosis and activation of the mitochondrial apoptotic pathway induced by excessive ROS (Wang et al., 2020). Nrf-2 serves pivotal functions in modulating inflammatory responses, oxidative stress, and the synthesis, metabolism, and degradation of proteins, lipids, and carbohydrates (Farkhondeh et al., 2020). Normally, Nrf-2 is broadly expressed in a large number of tissues including the testis and epididymis, resulting in preventing disruption of spermatogenesis due to oxidative stress (Nakamura et al., 2010). Based on some studies, the effects of supplementation on increasing Nrf-2 inducing Nrf-2 translocation and activating its target genes involved in cellular responses against ROS (Rungratanawanich et al., 2018).

Notably, no prior studies have assessed the effects of GO on behavioral stress and male reproductive parameters in CRS rats. While our study provides significant scientific evidence, certain limitations should be acknowledged. The small sample size in each study group might have yielded insufficient data for definitive conclusions. Additionally, the follow-up period's duration warrants consideration in future investigations. Furthermore, exploring the efficacy of low and high dosages of GO is a crucial aspect of future research endeavors.

The results indicated that GO supplementation can protect testes tissue against oxidative stress, apoptotic, hormonal, and histopathological alterations due to restraint stress. Thus, GO treatment can lead to recovery from male reproductive complications through regulating oxidative stress and apoptotic pathways. GO can be regarded as a beneficial substance for male infertility disorders. Further studies should be conducted to comprehend the molecular mechanism of testicular injury due to restraint stress and the possible capability of GO therapy or other substances to alleviate such effects on male infertility.
